# Fundamentals of Force-Controlled Friction Riveting: Part II—Joint Global Mechanical Performance and Energy Efficiency

**DOI:** 10.3390/ma11122489

**Published:** 2018-12-07

**Authors:** Gonçalo Pina Cipriano, Lucian A. Blaga, Jorge F. dos Santos, Pedro Vilaça, Sergio T. Amancio-Filho

**Affiliations:** 1 Institute of Materials Science, Joining and Forming, BMVIT Endowed Professorship for Aviation, Graz University of Technology, 8010 Graz, Austria; goncalo.pinacipriano@tugraz.at; 2 Department of Mechanical Engineering, School of Engineering, Aalto University, 02150 Espoo, Finland; pedro.vilaca@aalto.fi; 3 Helmholtz-Zentrum Geesthacht, Centre for Materials and Coastal Research, Institute of Materials Research, Materials Mechanics, Solid State Joining Process, 21502 Geesthacht, Germany; lucian.blaga@hzg.de (L.A.B.); jorge.dos.santos@hzg.de (J.F.d.S.)

**Keywords:** friction, riveting, hybrid structures, joining, response surface

## Abstract

The present work investigates the correlation between energy efficiency and global mechanical performance of hybrid aluminum alloy AA2024 (polyetherimide joints), produced by force-controlled friction riveting. The combinations of parameters followed a central composite design of experiments. Joint formation was correlated with mechanical performance via a volumetric ratio (0.28–0.66 a.u.), with a proposed improvement yielding higher accuracy. Global mechanical performance and ultimate tensile force varied considerably across the range of parameters (1096–9668 N). An energy efficiency threshold was established at 90 J, until which, energy input displayed good linear correlations with volumetric ratio and mechanical performance (R-sq of 0.87 and 0.86, respectively). Additional energy did not significantly contribute toward increasing mechanical performance. Friction parameters (i.e., force and time) displayed the most significant contributions to mechanical performance (32.0% and 21.4%, respectively), given their effects on heat development. For the investigated ranges, forging parameters did not have a significant contribution. A correlation between friction parameters was established to maximize mechanical response while minimizing energy usage. The knowledge from Parts I and II of this investigation allows the production of friction riveted connections in an energy efficient manner and control optimization approach, introduced for the first time in friction riveting.

## 1. Introduction

A current concern in industry is the compromise between the benefits of using lightweight materials and how to integrate these into larger multi-material designs. The wider the range of possible joining technologies to perform hybrid connections, the less compromising or restricted the usage of these materials might be. The more traditional and well-established methods to perform connections between different material classes are mechanical fastening [1] and adhesive bonding [2].

Given existing limitations related to the use of more conventional methods to perform connections (referred to in Part I of this work [3] and in References [2,4,5]) and the need to further push the boundaries on new design solutions and methodologies, several alternative joining technologies have been recently developed. Studies into how some of these hybrid joining technologies would perform under mechanical loading can be found in the literature. Abibe et al. [4] investigated the mechanical behavior of hybrid staked joints, performed using aluminum alloy AA2024-T351 and a 30% short-glass-fiber-reinforced polyamide 6,6. In their investigation, the failure of single-lap joints resulted from the bearing of the deformed polymeric stake against the inner wall of the pre-drilled feature on the metallic component, leading to both net tension and rivet pullout failure modes. Goushegir et al. [5] studied the mechanical performance of single lap joints produced by friction spot joining of AA2024 and carbon-fiber-reinforced poly(phenylene sulfide). Their work assessed the influence of the process parameters on the ultimate lap shear force and established a predictive analytical model, via a full-factorial design of experiments and analysis of variance (ANOVA). For a recent and comprehensive overview of friction-based joining processes for polymer-metal hybrid structures, please refer to Reference [6].

The present investigation focuses on evaluating the global mechanical performance of joints produced by friction riveting (FricRiveting), using polyetherimide (PEI) and AA2024-T351. Friction riveting was patented by Helmholtz-Zentrum Geesthacht [7] as a technique to produce both similar and dissimilar, polymer and hybrid polymer or composite-metal overlapping connections. The process has been reported to have successfully joined several material combinations. Initial studies on AA2024-T351/PEI joint formation and mechanical performance were performed by Amancio-Filho et al. [8], who produced and mechanically tested the joints by a quasi-static rivet pullout setup, observing several distinct types of failure. Failure throughout the rivet was achieved for some of the joining conditions tested [9]. Similar results were reported by Rodrigues et al. [10] for AA2024-T351/polycarbonate joints. By assessing joint formation measurements, they established a volumetric ratio for the plastically deformed rivet tip, plotted along with the maximum tensile force obtained from the quasi-static testing prior to failure. This analysis resulted in a relatively good correlation between the volumetric ratio and the load achieved. The increase of the former led also to an increase tendency displayed by the latter. This ratio, earlier introduced by Blaga et al. [11], establishes a simplified ratio between the plastically deformed rivet tip and the polymeric volume above it offering mechanical resistance to a rivet-pullout solicitation. In both works, this determined ratio had considerable scatter against the joint ultimate tensile force (UTF). These derived from the limitations of this ratio when a wide range of deformed rivet tip geometries is considered.

In the first part of the present work, by Cipriano et al. (Part I) found in Reference [3], the AA2024-PEI joints were produced, using a force-controlled, time-limited process variant of friction riveting. The resulting joint formation—the plastically deformed shape of the metallic rivet tip—was studied. Correlations between the joining process parameters and the resulting joint formation were established. Predictive statistical models were developed and reported for the following responses: rivet penetration depth; maximum width of the deformed rivet tip; and rivet anchoring depth. Building on this knowledge, the present work (Part II) aimed to evaluate the global mechanical properties of the exact same joints. A response surface methodology was used to statistically evaluate the response object of study, the UTF, and establish a predictive analytical model, with the objective of determining the expected mechanical behavior based on the joining process parameters. The mechanical energy input used to produce the joints, was evaluated along with the quasi-static joint mechanical performance and a concept of energy efficiency was established. Furthermore, an updated volumetric ratio calculation was proposed, to better take into consideration the wide range of rivet plastic deformation shapes and anchoring performance. This ratio was the basis to estimate joint global mechanical performance, based solely on joint formation. Finally, an optimized range of process parameters was defined for maximizing the UTF, while aiming to minimize the energy used.

## 2. The Process

Friction riveting (FricRiveting) is an alternative friction-based mechanical fastening method, combining principles from both conventional mechanical fastening and friction welding. FricRiveting can be performed using several setup configurations, such as single rivet and single polymeric plate (point-on-plate joints), and polymer–polymer or polymer–metal overlap joints [8]. The metallic rivet can be used either with a plain featureless surface or with different profiles, such as threaded [8] and hollow rivets [12]. The main connection mechanism of this process is mechanical interlocking, achieved between the plastically deformed rivet tip and the polymeric component enveloping it (i.e., through rivet anchoring). The rotating rivet is pressed into the polymer/composite, and given the local temperature increase during the process, it plastically deforms, assuming an axisymmetric anchor-shaped geometry and consolidating under pressure. A more detailed process description is given in Part I [3]. For this work, the user-defined process parameters are: rotational speed (RS); friction time (FT); friction force (FF); forging time (FoT); and forging force (FoF). The friction parameters are applied during the friction phase of the process, while the rivet is rotating and being inserted. After this friction phase the rotation is reduced to zero and a forging phase may take place, being defined by the forging parameters, force and time. Both friction and forging forces are axial forces applied to the rivet. Further detailed descriptions of the process and its configurations can be found in the literature [6,8] and in Part I of this work [3].

## 3. Materials and Methods

### 3.1. Base Materials

The materials used in the present work were polyetherimide (PEI) and AA2024-T351. Polyetherimide is a high-performance thermoplastic developed by Wirth et al. [13]. It is characterized by an elevated glass transition temperature (T_g_) at 215 °C [14]. Its mechanical behavior is in accordance with Hooke’s Law, having an elastic modulus that decreases by about 50% when at temperatures from 170 °C to 190 °C [15]. This engineering thermoplastic also meets automotive and aircraft industries’ specific requirements regarding flame resistance and smoke evolution [16]. Table 1 shows some of the properties characterizing this material. 

The polymeric joining parts, 70 mm × 70 mm, were machined from extruded PEI plates of 13.4 mm in nominal thickness, supplied by Quadrant Engineering Plastic Products, Lenzburg, Switzerland. The plain metallic rivets used for this work were produced out of extruded AA2024-T351 rods, having a length of 60 mm and a diameter of 5 mm. This alloy is characterized by high mechanical strength and is widely used for aircraft structural and fuselage applications, as well as for mechanical fasteners, making it very attractive for process developments of the present work nature. The properties of main interest for this alloy are shown in Table 2. For a more detailed description of the materials used, please refer to Part I [3].

### 3.2. Joining Procedure

**Figure 1:**
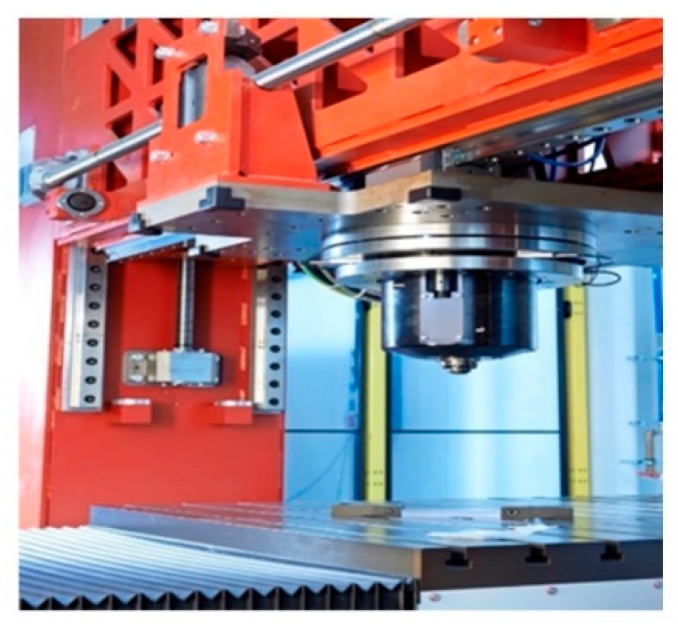
RNA custom equipment. (Photo: Helmholtz-Zentrum Geesthacht/Rasmus Lippels).

The joints tested in this investigation were produced in a customized FricRiveting gantry equipment (RNA, H. Loitz-Robotik, Hamburg, Germany). The joining equipment had a maximum axial load capacity of 24 kN and a maximum rotational speed of 21,000 rpm. The equipment allowed process on-line monitoring and the determination of the mechanical energy being used, with integrated sensors, namely assessing position, force, and torque. The equipment is shown in Figure 1.

The user-set process parameters for this force-controlled variant were: rotational speed (RS); friction force (FF); forging force (FoF); friction time (FT); and forging time (FoT). Table 3 presents the joining parameter ranges used.

The joining parameter combinations were set by Cipriano et al. in Part I [3] via a central composite design. The selection of the joining parameters was intended to promote a wide range of plastic deformation on the metallic rivet tip, aiming for providing an understanding of the energy ranges necessary to achieve a certain level of rivet mechanical anchoring in the polymeric part. Hence, correlating the energy input range and resulting rivet plastic deformation, with the global mechanical performance assessed in Part II of the work.

### 3.3. Non-Destructive Testing of Joint Formation

**Figure 2:**
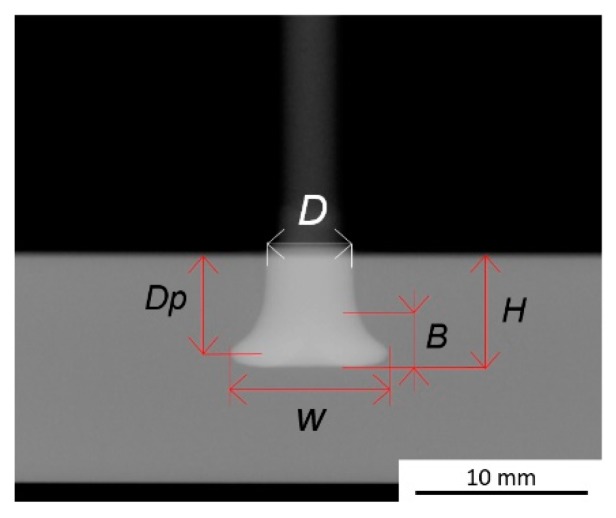
X-ray tomography joint formation measurements: rivet penetration depth (H); maximum width of the deformed rivet tip (W); depth until the maximum width of the rivet tip, or anchoring depth, (Dp); the height of the deformed rivet tip (B); and with original rivet diameter displayed (D).

The joint formation (i.e., the plastically deformed rivet tip geometry) was investigated in Part I [3], through X-ray tomography, exemplified in Figure 2.

Previous studies demonstrated the correlation between a volumetric ratio and the global mechanical performance of the joint [6,8,9]. The volumetric ratio establishes a simplified quotient between the volume of the plastically deformed rivet and the volume of polymer offering mechanical resistance to a rivet-pullout action. The volumetric ratio (VR) is determined by Equation (1):

(1) $$\mathrm{VR}=\frac{\left(H-B\right)\times \left(W^{2}-D^{2}\right)}{H\times W^{2}},\;\left[0-1\right]$$

where H is the penetration depth, B the deformed tip height (a dimension measured from the beginning of changes in the original rivet diameter, D, until the bottom of the deformed rivet tip, Figure 2), W the maximum deformed width of the rivet tip, and D the original rivet diameter. 

**Figure 3:**
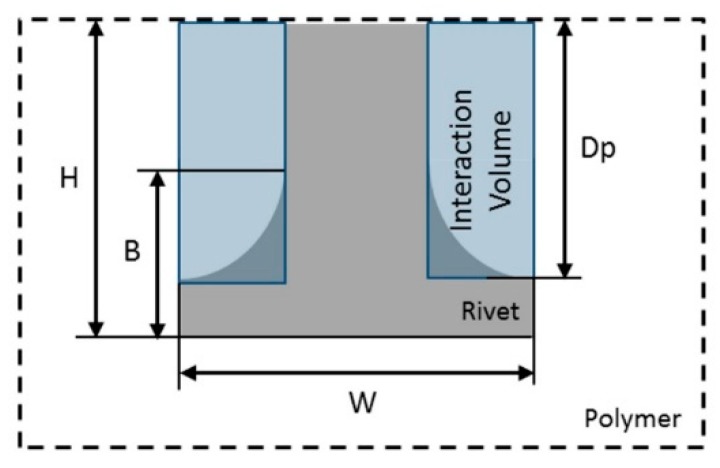
Schematic representation of joint formation geometrical measurements on a bell-shaped deformed rivet tip.

In the present work, an improved volumetric ratio assessment is proposed and compared with the previous approach. For distinguishing purposes, the updated volumetric ratio will be referred to as VR(U). This modification arose from the need to better assess the differences observed in joint formation and rivet tip shape, over a wide range of parameters reported in Part I [3] and further discussed in the results chapter. The VR(U) is expressed by Equation (2),

(2) $$\mathrm{VR}\left(U\right)=\frac{\left(W^{2}-D^{2}\right)\times D_{p}}{H\times W^{2}},\;\left[0-1\right]$$

where the new term Dp is used, representing the anchoring depth (i.e., the depth until the maximum width of the rivet tip), differing from the measure previously used based on B. Figure 3 schematically illustrates the limitation of the previous VR calculation procedure. For bell-shaped deformations of the rivet tip, using the B parameter leads to a considerable reduction of the polymeric interaction volume being considered. While by using Dp in the proposed VR(U) equation, a closer to reality and more robust estimation across a wide range of rivet tip deformations and geometries can be achieved, since Dp corresponds to the depth up to the maximum width of the deformed rivet tip. The limit cases where VR and VR(U) are equal to zero, entails that no rivet deformation has occurred, i.e., no interaction volume is present (Figure 3), and W has the value of the original rivet diameter, D. For both VR and VR(U) to achieve a value of one, some limit conditions must be met. The initial value of the diameter, D, would necessarily tend to the value of zero, with a W higher than D, for both ratios. For VR a B value close to zero would also need to be observed. In the case of VR(U), Dp would also tend to the same value as H.

### 3.4. Mechanical Performance

**Figure 4:**
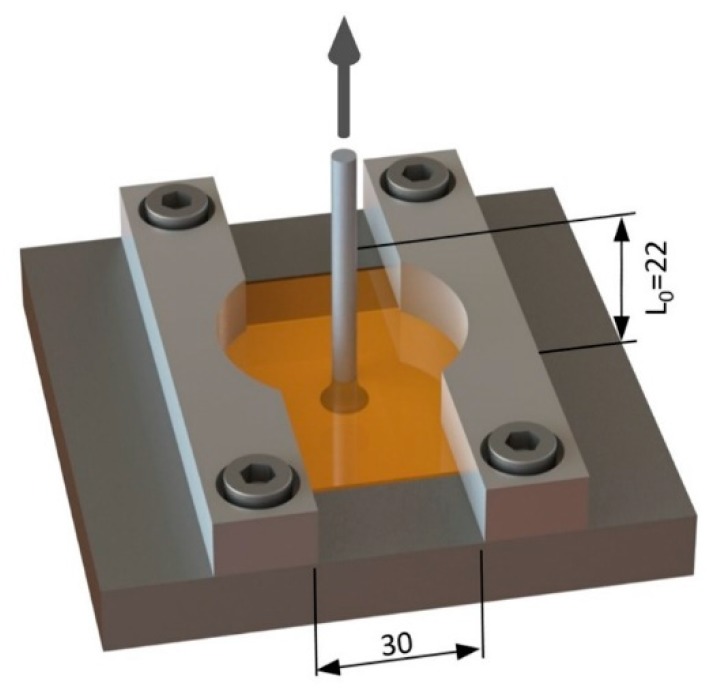
T-Pull testing set-up schematic representation (dimensions in millimeters).

To evaluate the global mechanical performance of the specimens produced, a quasi-static pullout tensile testing set-up was used (adapted from ISO 6892 [19]). The tests were conducted at room temperature using a Zwick/Roell 1478 universal testing machine (Zwick/Roell, Ulm, Germany) equipped with a 100 kN load cell. A customized clamping adapter, illustrated in Figure 4, was used to distribute the load over the polymeric plate. The specimens were tested at a rate of 1 mm/min and room temperature conditions, with a grip distance L_0_ of 22 mm. 

### 3.5. Energy Input

The energy input values used to produce the specimens for this work were calculated and reported by Cipriano et al. in Part I [3], using Equation (3). This equation considers the total mechanical energy input, E_M_, applied for friction-based processes, involving both metallic material [20] and thermoplastic [21].

(3) $$E_{M}=E_{f}+E_{d}=\int M\cdot \omega \cdot \mathrm{dt}+\int F\cdot \vartheta \cdot \mathrm{dt}\;\left[J\right]$$

The first term refers to the frictional energy (E_f_) resulting from torque (M) and rotational speed (ω). The second estimates the deformational component (E_d_), from axial force (F) and plunging rate of the metallic rivet (ϑ). The results previously reported on the energy input (Part I [3]) will sustain correlations and discussions between the energy used and the obtained global mechanical performance. 

### 3.6. Statistical Analysis of the Mechanical Performance Results

By using a design of experiments (DoE), Cipriano et al. (Part I) [3] determined the joining parameter combinations expected to yield a wide range of joint formation, and so, resulting in a large range of UTF. A central composite design (CCD) was used in Part I [3] to define the joining parameter test matrix. This is a second order design capable of generating response surfaces [22,23]. In the present work, the influence of the process parameters (RS, FT, FoT, FF, and FoF) on the UTF response was quantified and a predictive reduced regression model was established. This regression model was generated with a stepwise backward elimination procedure, considering an alpha-to-remove value of 0.05. By this method, all the potential terms of the model are considered at first, being the least significant term eliminated on each step; this iteration process is carried out up to the point at which no factor has a *p*-value above the defined alpha (i.e., being statistically significant). The model was validated by producing and testing additional joints with different parameter sets from the original design points, within the same parameter window.

## 4. Results and Discussion

### 4.1. Volumetric Ratio Assessment

As described in Section 3.3, the volumetric ratios, VR and VR(U), were determined by making use of the measurements on joint formation, published in Part I [3]. The calculated values are shown in Table 4. As previously discussed, VR gives an indication of the expected mechanical performance (UTF) of a given joint [10,11]. 

**Figure 5:**
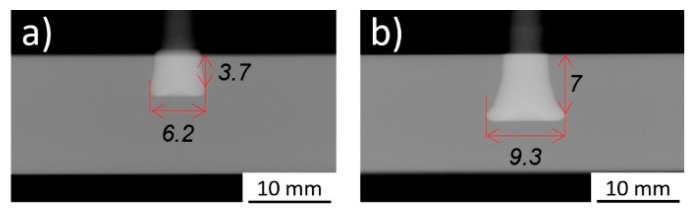
X-ray tomography of: (**a**) Condition 1 (E_M_ = 24 J; RS = 18,000 rpm; FT = 1.6 s; FoT = 1 s; FF = 2000 N; FoF = 5100 N); (**b**) Condition 4 (E_M_ = 77 J; RS = 20,000 rpm; FT = 2 s; FoT = 1 s; FF = 2000 N; FoF = 5100 N).

The joints which yielded both the lowest and the highest VR(U), Conditions 1 (VR = 0.14/VR(U) = 0.28) and 4 (VR = 0.27/VR(U) = 0.66) are shown in Figure 5. It is clear that relevant differences in rivet tip deformation were achieved. These conditions were produced with different joining parameters, which resulted in different total energy inputs (Condition 1: E_M_ = 24 J; Condition 4: E_M_ = 77 J). The influence of the energy input on joint formation will be addressed in the following sections.

### 4.2. Global Mechanical Performance

The global mechanical performance of the joints was assessed by the procedure described in Section 3.4. The UTF and mechanical energy input, E_M_ [3], values achieved during testing are presented in Table 5.

**Figure 6:**
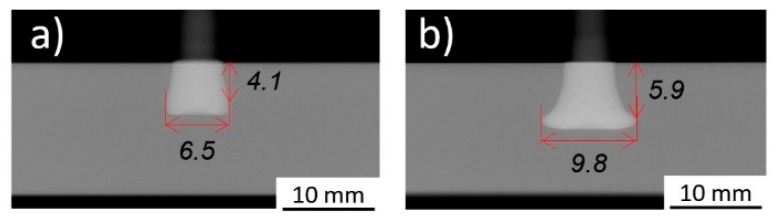
X-ray tomography of: (**a**) Condition 33 (RS = 19,000 rpm; FT = 1.8 s; FoT = 1.5 s; FF = 1500 N; FoF = 4500 N); (**b**) Condition 36 (RS = 19,000 rpm; FT = 1.8 s; FoT = 1.5 s; FF = 2500 N; FoF = 5700 N).

The highest UTF was achieved for Condition 36 (E_M_ = 86 J), with a value of 9668 N. The lowest UTF value was obtained for Condition 33 (E_M_ = 38 J), 1096 N. The latter condition is characterized by a very small rivet tip plastic deformation inside the polymer, consequence of the smaller E_M_. Hence, a lower strength mechanical anchoring resulted between the plastically deformed rivet tip and the polymeric plate. As can be seen in Figure 6a, this joining condition induced only a slight change of the rivet diameter at the tip (W = 6.5 mm) in comparison to the original rivet diameter (5 mm).

As opposed to what is seen in Figure 6a, Condition 36 joined with higher FF and FoF (refer to Figure 6 caption or Table 2 in Part I [3]) in Figure 6b, yielded the highest UTF. In this case, the observed deformation sustained by the rivet is considerably higher, resulting in a bell-shaped rivet tip. The mechanical anchoring of the deformed rivet tip inside the polymer increases the UTF by an 8.8 factor, when compared with Condition 33, from 1096 N to 9668 N. There is a significant increase in the deformed rivet tip diameter (W = 9.8 mm), while the rivet penetrates deeper into the polymer (H = 5.9 mm). Consequently, a greater polymeric interaction volume resists the pullout mechanical solicitation during testing. The higher interaction volume is demonstrated by the increase of VR(U) for Condition 36 (0.63) by a factor of 1.97 from that of Condition 33 (0.32). From this example, one might consider that by increasing the energy input used, it would invariably result in a higher VR(U) and consequently in a higher mechanical performance of the joint. Nonetheless, after a certain energy level is reached, the increasing plastic deformation will result in a decrease of VR(U). In the coming sections, the corroborated effect of energy input and other factors, such as geometrical shape and features of the anchoring zones, on the anchoring performance will be discussed.

**Figure 7:**
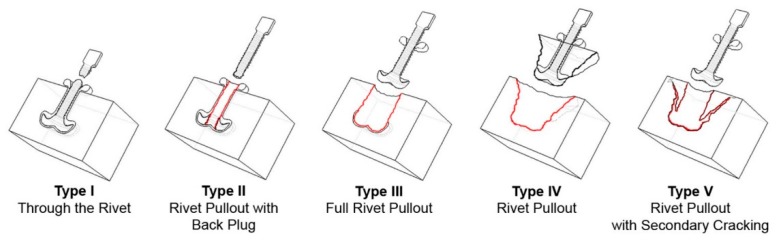
Current failure modes of friction riveted joints reported in the literature (Reprinted from *J. Mater. Process Technol.* [10]).

Different joint failure types were observed for the tested specimens. Figure 7 schematically represents the current classification of the several failure modes reported in the literature [10] for metallic-insert friction riveted joints. 

The fractures of the conditions tested in this work were in accordance with those previously reported in the literature [8,10,24] and are summarized in Table 6.

**Figure 8:**
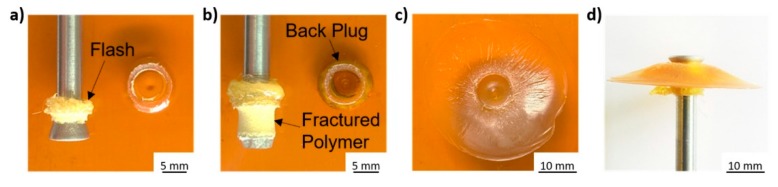
Examples of failure modes observed: (**a**) Condition 7 (RS = 18,000 rpm; FT = 2 s; FoT = 2 s; FF = 2000 N; FoF = 5100 N), full rivet pullout failure; (**b**) Condition 30 (RS = 19,000 rpm; FT = 2.2 s; FoT = 1.5 s; FF = 2500 N; FoF = 4500 N), rivet pullout with back plug; (**c**) Condition 13 (RS = 18,000 rpm; FT = 1.6 s; FoT = 2 s; FF = 3000 N; FoF = 5100 N), polymeric plate, rivet pullout failure; (**d**) Condition 13, side view of rivet and detached polymer, rivet pullout failure.

Although an early initial ductile necking of the exposed rivet was verified in Condition 36, none of the tested conditions displayed a Type I failure mode. Both full rivet pullout (Type III) and rivet pullout (Type IV) failures, occur when the polymer is not capable of sustaining the mechanical solicitation [8]. The difference between these two failure types is that, in Type III, there is no fractured polymer from the polymeric interaction volume being removed with the rivet, although the expelled flash does stay attached to the rivet shaft, as seen in Figure 8a (Condition 7, E_M_ = 65 J). Here, the low deformation of the rivet tip allows it to be pulled by radially deforming the polymeric interaction volume, as it slides out. In failure Type II (rivet pullout with back plug), the interaction volume can sustain the mechanical solicitation. In this case the failure takes place on the rivet deformed tip, leaving a back plug of metal inside the polymer. This occurs when the resistance of the transition metallic area between main rivet body and the deformed tip is inferior to that offered by the polymeric interaction volume above it [10]. An example of Type II is seen in Figure 8b, for Condition 30 (E_M_ = 136 J). Rivet pullout (type IV) corresponds to the joints which yielded the highest UTF in this study. In these cases, the deformed rivet tip can withstand the solicitation and not fail on the metal, hence, not leaving the back plug observed in failure Type II. The deformation for these rivet pullout cases, is sufficient to promote a good mechanical anchoring inside the polymer, forcing it to bare the mechanical solicitation up to final failure. Figure 8c,d, represent the rivet pullout failure observed for Condition 13 (E_M_ = 78 J).

**Figure 9:**
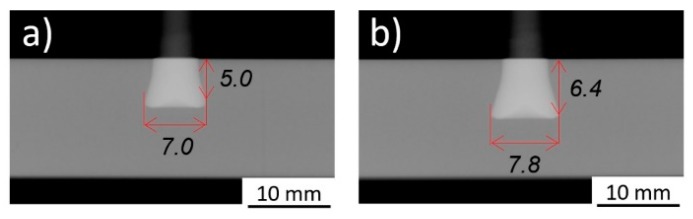
X-ray tomography of: (**a**) Condition 6 (RS = 20,000 rpm; FT = 1.6 s; FoT = 2 s; FF = 2000 N; FoF = 5100 N); (**b**) Condition 7 (RS = 18,000 rpm; FT = 2 s; FoT = 2 s; FF = 2000 N; FoF = 5100 N).

From the volumetric ratio assessment in the previous section, the VR(U) of Conditions 1 and 4 (0.28 and 0.66, respectively) are in accordance with the expected indication they give on UTF, as Condition 1 yielded a UTF of 1776 N and Condition 4 of 9619 N. The same was not observed for the rivet mechanical anchoring performance assessed by VR. For instance, Condition 4 (UTF = 9619 N), with a calculated VR of 0.27, was far from the maximum VR value observed of 0.48 for Condition 30, although the latter has a smaller UTF (8643 N). These results suggest that the state-of-the-art VR equation has limitations when comparing different deformation magnitudes. This is clearer when comparing Conditions 6 (E_M_ = 36 J) and 7 (E_M_ = 65 J), despite having the same VR of 0.21, demonstrate different VR(U), 0.45 and 0.55, respectively. As can be seen from X-ray tomography images (Figure 9), although the overall geometry is similar in both joints, both the rivet penetration and deformation of Condition 7 are greater than that of Condition 6. In this comparison, calculated VR(U) values are proportional to UTF, whereby Condition 7 is stronger than Condition 6 (UTF = 6256 N and UTF = 3897 N, respectively). Therefore, the modified rivet mechanical anchoring estimation by VR(U) (Equation (2)) seems to allow for a better fitting with the joint mechanical performance.

**Figure 10:**
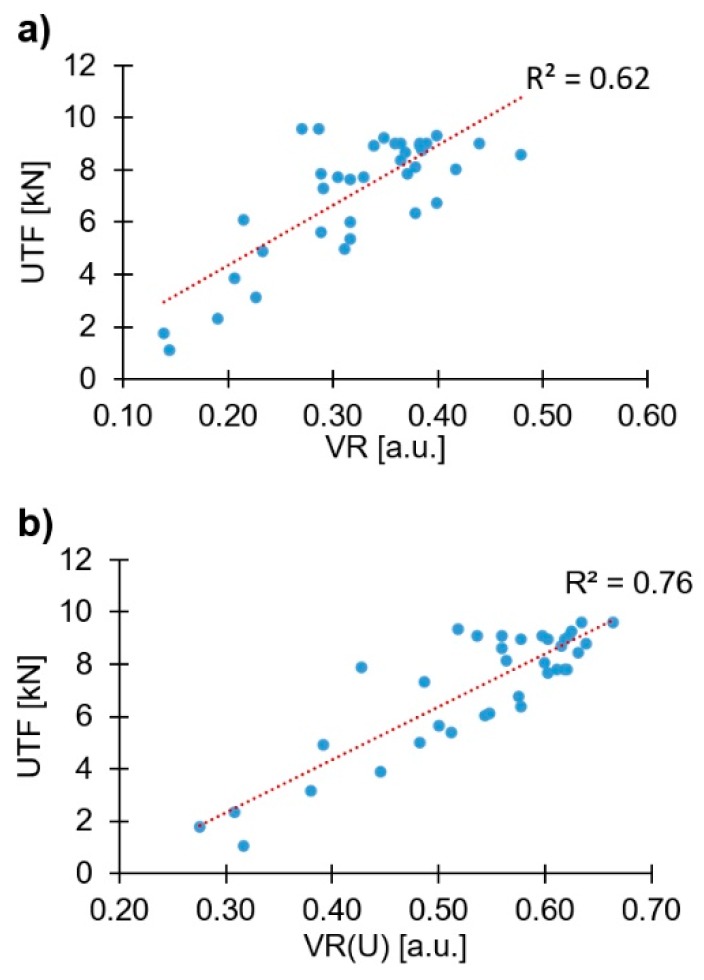
UTF—volumetric ratio plots: (**a**) VR; (**b**) VR(U).

The correlation between both VR and VR(U), with the global mechanical performance (UTF) of the joints produced, can be seen in Figure 10.

Although the data demonstrates the tendency of linear proportionality of the volumetric ratio with the UTF, as reported in the literature for the VR [10], an improvement to this direct correlation (based on the value of the correlation performance parameter R^2^) is observed when considering VR(U). This further supports the assumption that the VR(U) is a more accurate method of assessing the rivet mechanical anchoring and estimating joint mechanical performance, as it better takes into consideration the variations in shape/geometry of the rivet tip. Thus, for the remainder of the analysis, only the VR(U) will be considered.

### 4.3. Energy Efficiency

**Figure 11:**
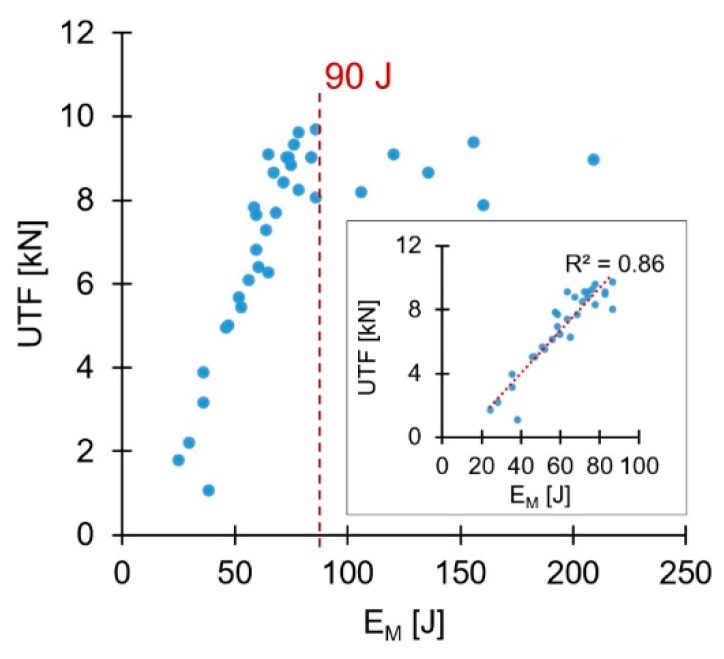
Correlation between mechanical performance and total energy input (E_M_). In detail, the correlation for the energy efficient range (E_M_ ≤ 90 J).

Energy input during friction riveting has been addressed in the literature [25], with some correlations being established with the joining parameters used. Nonetheless, no correlation between the energy input and mechanical performance has been analyzed. Therefore, a notion of energy efficiency, concerning the joint quasi-static mechanical performance, is then necessary. This could minimize the energy input and reduce costs (e.g., reduce power consumption, joining time.) when producing joints with higher mechanical performance. Hence, the total energy inputs (E_M_), previously published for the present conditions in Part I, were evaluated in terms of UTF. Figure 11 presents the correlation between the total energy input and the respective UTF values.

**Figure 12:**
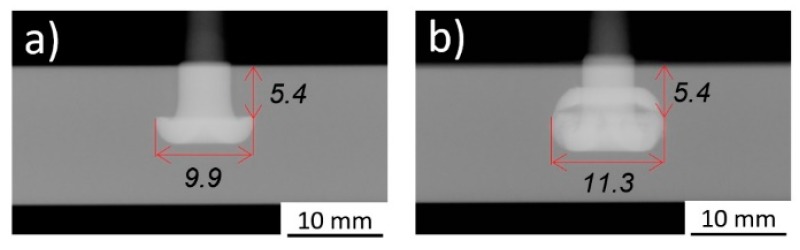
X-ray tomography of: (**a**) Condition 15 (RS = 18,000 rpm; FT = 2 s; FoT = 2 s; FF = 3000 N; FoF = 3900 N); and (**b**) Condition 12 (RS = 20,000 rpm; FT = 2 s; FoT = 1 s; FF = 3000 N; FoF = 3900 N).

A relatively linear initial tendency of increasing UTF with the increase of the total energy input is evident. Also, relatively clear, is the level of energy input at which this correlation ceases to be valid (E_M_ ≈ 90 J), with UTF reaching a relative plateau. As mentioned in Part I, above a certain level of energy input, the resultant deformation of the rivet tip is considered over-deformation. This is characterized by a small Dp, resulting from a premature increase of W (detailed in Figure 20, Part I [3]). Condition 15 (E_M_ = 120 J), Figure 12a, illustrates a small amount of over-deformation, contrasting with the bell-shaped plastically deformed rivet tip seen in Figure 5b and Figure 6b. Figure 12b shows Condition 12 (E_M_ = 155 J), exemplifying a considerably over-deformed rivet tip. In these particular cases of excessive deformation, VR was more sensitive to plastic deformation changes than VR(U). Despite this fact, as shown in Figure 10, the accuracy of VR(U) remains higher than that pf VR, across the rivet plastic deformation range observed in this study. This is further accentuated when the energy efficient threshold is imposed (90 J), Figure 11.

**Figure 13:**
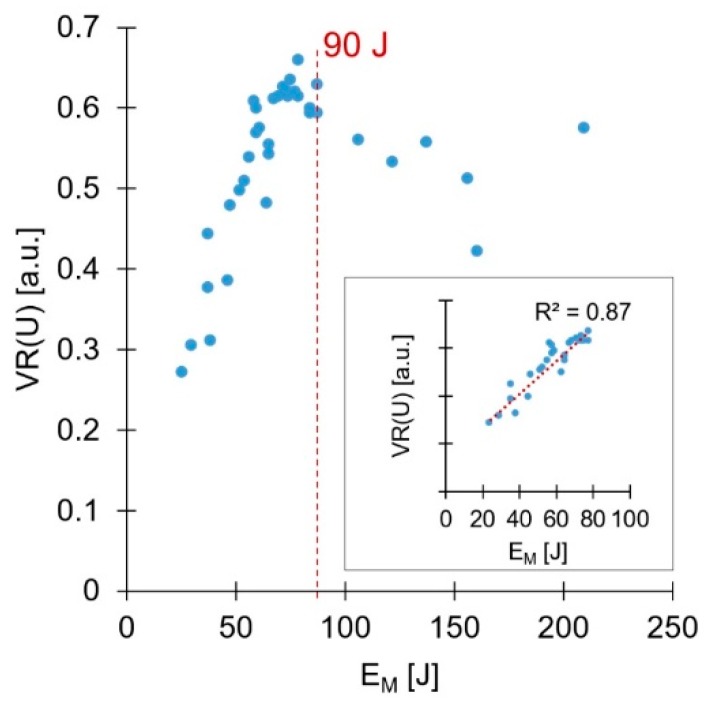
Correlation between improved volumetric ratio, VR(U), and total energy input (E_M_). In detail, correlation for the energy efficient range (E_M_ ≤ 90 J).

Over-deformation of the rivet tip is considered negative since it does not inherently lead to a higher joint mechanical performance. By the correlation established in Figure 11, the UTF of Condition 12 (9362 N) could be achieved by a joint produced with a much lower energy input (E_M_ ≈ 80 J). Figure 13 shows an energy efficiency perspective over the process before any mechanical testing, by assessing VR(U). Similar to the discussion regarding UTF, an energy efficient joint formation threshold (regarding VR(U)) can also be established. Therefore, using energy inputs higher than the defined thresholds means unnecessary consumption of resources. Therefore, this should be taken into consideration when defining joining parameters with a targeted UTF value, to minimize production costs.

### 4.4. Influence of the Process Parameters on Mechanical Performance

The quasi-static mechanical performance (UTF) of the produced joints was statistically investigated. Response surface methodology was used to determine the influence joining process parameters have on this mechanical response, as described in Section 3.6.

The first order parameters present in the reduced statistical model were RS, FT and FF. The second order parameters were FF × FF and FT × FT. Finally, the interaction FT × FF is also considered. The respective *p*-values of these parameters are presented in Table 7.

**Figure 14:**
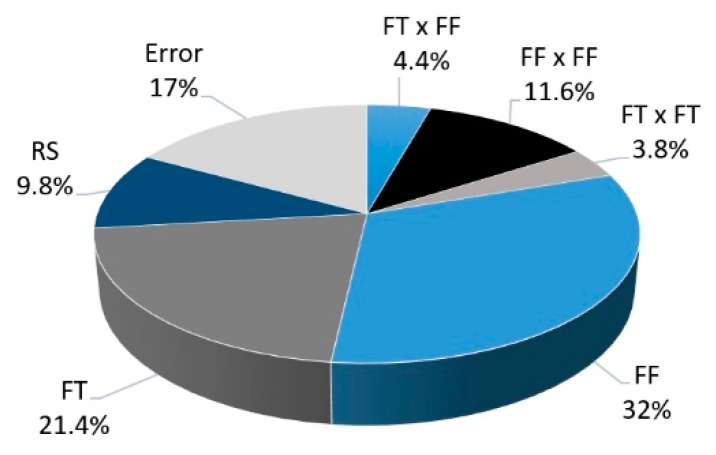
Relative contributions of the model factors to the response UTF.

The individual contributions of the terms present in the model and its total error are shown in Figure 14. The largest contribution (32%) to the achieved UTF comes from a linear term, the FF parameter. One quadratic term, FF × FF, also plays an important role with 11.6%.

From the statistical analysis, FoF and FoT are not part of the model, having *p*-values larger than 0.05 (Table **7**). This behavior may relate to the investigated joining parameters windows, as these may be narrow for FoF and FoT to promote considerable variance on the mechanical performance across the entire selected range, independently of the relatively wide range of resulting rivet plastic deformation in this work.

The reduced regression equation obtained for this predictive model is shown below (Equation (4)) in parameter-coded levels [−2; 2].

(4) $$\begin{array}{c} UTF=8016+887\;RS+1310\;FT+1602\;FF-\\ \left(478\;FT\times FT+835\;FF\times FF+726\;FT\times FF\right) \end{array}$$

**Figure 15:**
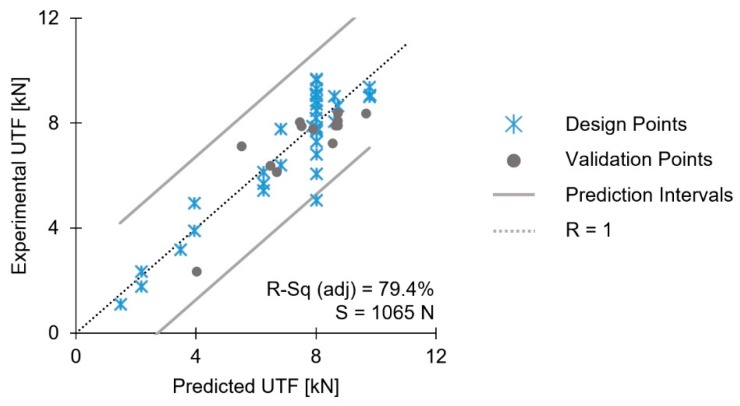
Validation diagram for the reduced model of UTF.

The model-predicted values for UTF were correlated with those obtained experimentally in Figure 15. In this validation plot, it is visible that the majority of the data points fall within the prediction limit lines (solid grey), within which the model can predict a single response observation [26]. A set of 13 additional validation joints supported this trend.

The explanatory power of the UTF model—the adjusted R-sq—was 79.4%. Moreover, this model shows a predicted R-sq = 77.9% and standard error, S, of 1065 N.

**Figure 16:**
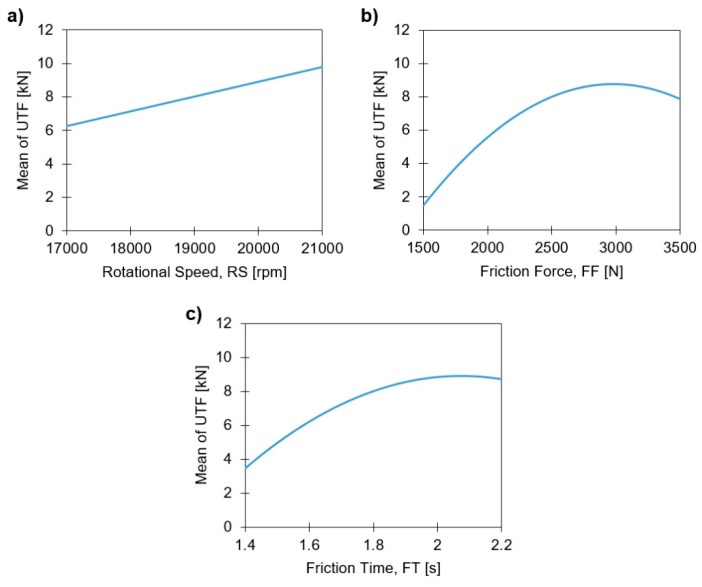
Main effects plots of linear model terms for the UTF response, with: (**a**) Rotational Speed; (**b**) Friction Force; and (**c**) Friction Time.

Figure 16 displays the influence the joining parameters have on UTF, using the main effects plots.

The only parameter that does not present complex effects is RS, with its increase resulting in higher UTF, given its linearly increasing contribution to the energy input. In other words, the RS promotes a higher rivet penetration, H, (refer to Figure 10a in Part I [3]), without reducing the polymeric interaction volume or negatively affecting the anchoring depth, Dp, as presented in Figure 19a, Part I. Both FF and FT display relatively similar curves over the studied range. In both cases the maximum UTF value is achieved at the upper quarter of the parameter range. Altmeyer et al. [25], have found that for friction riveting of titanium grade 3 with short-fiber-reinforced polyether-ether-ketone (PEEK), the UTF increased mostly due to the increase of RS, FT, and forging pressure (FoP) [27]. The high contribution of FF has been reported also in Part I of this investigation. Both FT and FF effects on the UTF become negative when close to the upper limits of the investigated parameter ranges. This can be explained by the occurrence of over-deformation on the metallic rivet tip, also reported in Part I, which is counterproductive towards the mechanical performance of the joint and not energy efficient, as demonstrated in Section 4.3.

**Figure 17:**
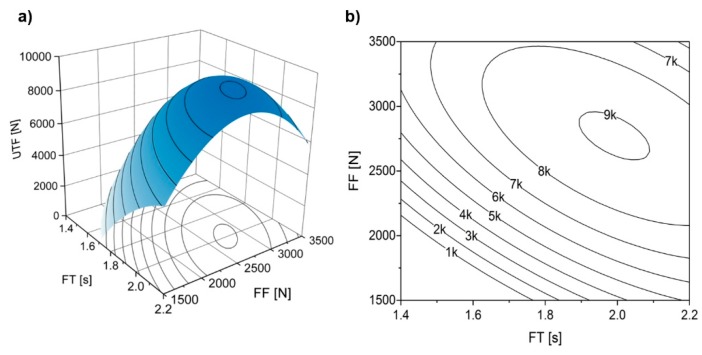
Effect of FT × FF two-way parameter interaction on UTF (in newton), (**a**) surface and (**b**) contour plots. RS set at 19,000 rpm, FoF at 4500 N, and FoT at 1.5 s.

The two-way interaction part of the statistical model (FT × FF) is depicted in the surface and contour plots of Figure 17. In order to assess this interaction, the remaining parameters (RS, FoT and FoF) were set at their respective middle range values [23].

It seems that only a small area (peak of the surface in Figure 17a) of both FT and FF ranges can maximize the UTF. This roughly elliptic area is located around FF values of 2750 N and FT around 2.0 s. Given the orientation of the curves (Figure 17b), smaller variations of FF than those of FT may result in UTF values outside the peak and optimal response region. This behavior was also seen in the main effects plots (Figure 16b,c), where effect of FF displayed a more pronounced curvature than FT. At low levels of both parameters, the resulting low energy input will not promote sufficient plastic deformation on the rivet tip to resist the pullout solicitation, hence the low UTF for this left lower quarter parameter region (e.g., Condition 1, seen in Figure 5, with a VR(U) = 0.28 and E_M_ = 24 J). In Figure 17b, following a 1:1 correlation between FF above 3000 N and FT higher than 2.0 s the UTF begins to gradually decrease to around 6000 N for maximum values of FF and FT. Since high levels of these parameters tend to produce over-deformation on the rivet tip, e.g., Condition 15, seen in Figure 12a. This is also in accordance to the proposed threshold of energy efficiency of 90 J (see Figure 11 and Figure 12 for reference). 

### 4.5. Summary of the Findings

In Part I of this work, the influence of the process parameters on the plastic deformation of the tip of the rivet was investigated. The energy input during the production of the joints was calculated and predictive reduced statistical models were established for the geometrical features of joint formation: rivet penetration depth, maximum width of the deformed rivet tip, and anchoring depth. An initial optimized range of parameters for joint formation was established with the aim of improving the mechanical performance of the joints. In the present Part II, the mechanical performance of the joints was assessed. Based on the UTF and the energy input, the energy efficiency of the joints was investigated. The anchoring efficiency estimation by volumetric ratio (VR) was amended and the proposed updated model was validated (VR(U)). The energy efficiency and UTF were then correlated with the joint formation, using VR and VR(U), with an improved accuracy in the case of the latter. 

**Figure 18:**
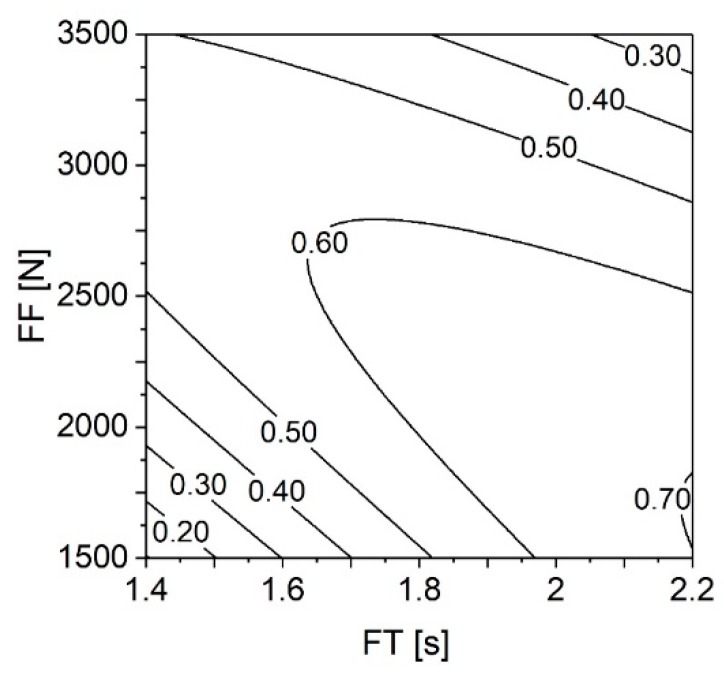
Contour plot for VR(U) correlated with FT and FF ranges. RS set at 21,000 rpm, FoT at 1.5 s, and FoF at 4500 N.

In Part I [3], resulting from the reduced statistical models, Figure 22 illustrated the influence of FT and FF on H, W, and Dp. A similar approach was now used for VR(U), across the ranges of these parameters (Figure 18).

This plot shows how the use of maximum values for both FT and FF (i.e., higher energy input) leads to a VR(U) lower than 0.30. This is almost as low as the VR(U) resulting from using minimum values of those same parameters (VR(U) < 0.20). The decline in VR(U) observed when using energy input values beyond the energy efficiency threshold (Figure 13) is a result of rivet over-deformation, characterized by a decrease of the polymeric interaction volume. This effect has been discussed in Section 4.3 (Figure 12) and in more detail in Part I (Section 4.3.3; Figure 20) [3] It is directly correlated with the notion of energy efficiency, as using an excessive energy input (E_M_ > 90 J) tends to result in a decrease of the mechanical performance of the joints. An example of this effect summarized in Table 8, is the correlation between VR(U), UTF, and E_M_ between Conditions 25 (RS = 19,000 rpm; FT = 1.8 s; FoT = 1.5 s; FF = 2500 N; FoF = 4500 N) and 34 (RS = 19,000 rpm; FT = 1.8 s; FoT = 1.5 s; FF = 3500 N; FoF = 4500 N), with the only different parameter being FF.

The total energy input calculated for Condition 25 is 43% of that used to produce Condition 34. The UTF of both conditions are in accordance with Figure 17b, for the respective FT and FF parameters. For both conditions, the plotted UTF values are close to reach the ellipsoidal 8000 N contour line. This demonstrates the energetic inefficiency of Condition 34, as the remnant 57% of the energy input used did not contribute to a significant improvement of the mechanical anchoring performance of the joint (UTF). 

**Figure 19:**
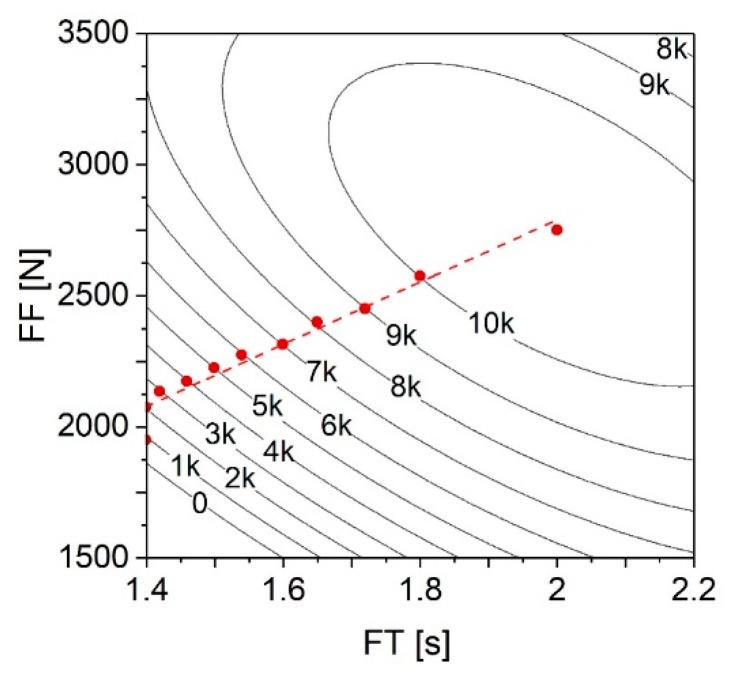
Contour plot for UTF (in Newton) correlated with the ranges of FT and FF. RS set at 21,000 rpm, FoT at 1.5 s, and FoF at 4500 N.

As shown in the previous section, RS has a linear increasing effect on the UTF (Figure 16a). Given this fact, in order to maximize the UTF response, Figure 19 displays a contour plot analogue to that of Figure 17b, now with RS set to its maximum (21,000 rpm). This allows to investigate how this increase of RS influenced the contour lines across the ranges of both FT and FF. The remaining parameters (FoT and FoF) were set to their central values (1.5 s and 4500 N, respectively), considering that these did not demonstrate a statistically significant effect on the UTF response.

As expected, the peak region where the UTF is maximized remains centered in the vicinity of the same point (FT = 2 s and FF = 2750 N), given the mentioned linear effect RS has on UTF (Figure 16a). However, this region increased both in area and in UTF, from 9000 N ≤ UTF < 10,000 N to 10,000 N ≤ UTF < 11,000 N.

From these discussions, an energetically efficient approach toward producing a strong joint with this combination of materials, can be accomplished by using the highest value of RS from the investigated range (RS = 21,000 N), central values of both FoT and FoF (FoT = 1.5 s; and FoF = 4500 N), with FT and FF being chosen along the marked dashed line in Figure 19 (FF = 1189.8 × FT + 403.6, SI units). This correlation between FF and FT has been determined by linear regression from the points (seen as red circles in Figure 19) which represent the local minimums of the respective contour lines and also the predicted maximum (FT = 2 s and FF = 2750 N). These points identify a minimum combination of FT and FF values, which yield the respective UTF. In this manner, the energy usage is optimized (i.e., minimized), while maximizing the corresponding mechanical performance.

## 5. Conclusions

The global mechanical performance of hybrid connections produced using force-controlled and time-limited friction riveting, was investigated in this second and final part of the study into the fundamentals of this process variant. The joints previously produced (using AA2024-T351 and polyetherimide) and studied in Part I [3] of the work—assessment of joint formation—were mechanically tested in Part II. The ultimate tensile force (UTF) of the joints (rivet pullout solicitation) was determined, ranging between 1096 and 9668 N. The knowledge on joint formation from Part I [11] allowed the assessment of the anchoring efficiency, using the previously established volumetric ratio (VR) and a revised improved calculation (VR(U)). The latter was demonstrated to have a more accurate correlation with the mechanical performance, across the observed deformations and respective VR(U) range (0.28–0.66). The influence and contributions of the process parameters, their quadratic effects and interactions, on the mechanical performance were assessed using response surface methodology and statistical analysis of variance. Friction force and friction time parameters displayed complex behavior across their ranges showing a curvature related to over-deformation of the rivet tip. Increasing rotational speed promoted a linear increase of the mechanical performance. Forging parameters did not display a statistically significant effect on the performance response for the investigated range of parameters.

The wide range of plastic deformation experienced by the rivet tip, led to a wide range of mechanical performance and different joint failure types: full rivet pullout; rivet pullout with back plug; and rivet pullout. The highest values of VR(U) (above 0.60) yielded the highest mechanical performances (9619 N), in joints with the deformed rivet tip having a characteristic overall bell-shaped geometry (e.g., Condition 36, Figure 6b). A maximum threshold of energy efficiency was established at 90 J. Above this value, the energy used no longer contributes toward the increase of mechanical performance, instead promoting a counterproductive over-deformation of the rivet tip, with decreasing anchoring efficiency. Bellow this threshold, the energy input was found to have good linear correlations both with the new proposed volumetric ratio (VR(U)), reaching an R-sq = 0.87, and the ultimate tensile force, reaching an R-sq = 0.86. The highest contributions to the UTF originated from the friction parameters: force (FF = 32.0%) and time (FT = 21.4%). Both demonstrating the influence of higher order effects. Neither forging force (FoF) nor forging time (FoT) were found to be statistically significant for the investigated joining range of parameters. The region of maximum mechanical performance was found to be centered on 2750 N for friction force and 2 s for friction time, yielding ultimate tensile forces above 10,000 N. An optimized correlation capable of maximizing the mechanical performance and simultaneously minimizing energy input, across the parameter ranges, was established (FF = 1189.8 × FT + 403.6, SI units). This allows the production of joints with pre-determined mechanical properties, without unnecessary expenditure of energy and material.

The conclusions of the present work, with the knowledge from Part I [3], allow for an estimation of the expected global mechanical performance, of friction riveted AA2024/polyetherimide hybrid point-on-plate joints, based on process parameters and mechanical energy input. Furthermore, the proposed volumetric ratio amendment, VR(U), improves the assessment of the anchoring efficiency.

## Figures and Tables

**Table 1:** Summarized polyetherimide (PEI) properties [17].

Property	Value
R_0.2_ (MPa)	129
E (MPa)	3500
Glass Transition Temp. (°C)	215
Thermal Conductivity (W/m·K)	0.24

**Table 2:** Summarized AA2024-T351 properties [18].

Property	Value
R_m_ (MPa)	427
R_0.2_ (MPa)	310
E (GPa)	72
Melting Temp. Domain (°C)	518–548
Sol. Heat Treat. Temp. (°C)	495
Annealing Temp. (°C)	256

**Table 3:** Process parameter ranges.

RS [rpm]	FT [s]	FoT [s]	FF [kN]	FoF [kN]
17,000–21,000	1.4–2.2	0.5–2.5	1.5–3.5	3.3–5.7

**Table 4:** Volumetric ratios of the produced joints.

Condition	VR	VR(U)	Condition	VR	VR(U)	Condition	VR	VR(U)
**1**	0.14	0.28	**13**	0.33	0.62	**25**	0.30	0.62
**2**	0.23	0.39	**14**	0.42	0.60	**26**	0.36	0.63
**3**	0.32	0.51	**15**	0.44	0.54	**27**	0.29	0.50
**4**	0.27	0.66	**16**	0.38	0.58	**28**	0.39	0.60
**5**	0.19	0.31	**17**	0.29	0.49	**29**	0.23	0.38
**6**	0.21	0.45	**18**	0.35	0.62	**30**	0.48	0.56
**7**	0.21	0.55	**19**	0.44	0.64	**31**	0.36	0.56
**8**	0.37	0.61	**20**	0.38	0.62	**32**	0.36	0.62
**9**	0.38	0.58	**21**	0.31	0.54	**33**	0.18	0.32
**10**	0.34	0.60	**22**	0.31	0.60	**34**	0.20	0.43
**11**	0.38	0.56	**23**	0.31	0.48	**35**	0.40	0.57
**12**	0.40	0.52	**24**	0.37	0.62	**36**	0.28	0.63

**Table 5:** Mechanical testing ultimate tensile force (UTF) results.

Condition	E_M_ (J)	UTF (N)	Condition	E_M_ (J)	UTF (N)	Condition	E_M_ (J)	UTF (N)
**1**	24	1776	**13**	78	8251	**25**	68	7741
**2**	46	4943	**14**	86	8046	**26**	71	8461
**3**	53	5427	**15**	120	9106	**27**	51	5689
**4**	77	9619	**16**	208	8996	**28**	83	9049
**5**	29	2202	**17**	63	7290	**29**	36	3166
**6**	36	3897	**18**	76	9304	**30**	136	8643
**7**	65	6256	**19**	74	8824	**31**	64	9098
**8**	57	7829	**20**	73	9033	**32**	73	9029
**9**	60	6391	**21**	56	6068	**33**	38	1096
**10**	83	9004	**22**	59	7663	**34**	159	7864
**11**	106	8192	**23**	47	5041	**35**	59	6811
**12**	155	9362	**24**	67	8701	**36**	86	9668

**Table 6:** Types of fracture obtained for the tested joints.

Failure Type	Condition	UTF Range [N]
Rivet pullout with back plug (Type II)	12, 19, 21, 23, 26, 30, 35	5041–9362
Full Rivet Pullout (Type III)	1, 2, 3, 5, 6, 7, 8, 9, 10, 11, 17, 18, 20, 22, 24, 25, 27, 29, 33	1096–9049
Rivet Pullout (Type IV)	4, 13, 14, 15, 16, 28, 31, 32, 34, 36	7864–9668

**Table 7:** Statistical significance (p-values) of reduced model factors.

Parameter	*p*-Value	Parameter	*p*-Value
**RS**	0	**FF × FF**	0
**FT**	0	**FT × FT**	0.017
**FF**	0	**FT × FF**	0.011

**Table 8:** Joint performance comparison.

Condition	VR(U)	UTF [N]	E_M_ [J]
25	0.62	7741	68
34	0.43	7864	159

## References

[1] Amancio-Filho S.T., Abibe A.B., Dos Santos J.F., Nicolais L., Borzacchiello A. (2012). Joining: Mechanical Fastening of Polymers, Composites, and Polymer–Metal Hybrid Structures. Wiley Encyclopedia of Composites.

[2] Amancio-Filho S.T., Dos Santos J.F. (2009). Joining of polymers and polymer-metal hybrid structures: Recent developments and trends. Polym. Eng. Sci..

[3] Pina Cipriano G., Blaga L., F. dos Santos J., Vilaça P., Amancio-Filho S. (2018). Fundamentals of Force-Controlled Friction Riveting: Part I—Joint Formation and Heat Development. Materials (Basel).

[4] Abibe A.B., Amancio-Filho S.T., dos Santos J.F., Hage E. (2013). Mechanical and failure behaviour of hybrid polymer–metal staked joints. Mater. Des..

[5] Goushegir S.M., dos Santos J.F., Amancio-Filho S.T. (2015). Influence of process parameters on mechanical performance and bonding area of AA2024/carbon-fiber-reinforced poly(phenylene sulfide) friction spot single lap joints. Mater. Des..

[6] Amancio-Filho S.T., Lucian-Attila Blaga L.-A. (2018). Joining of Polymer-Metal Hybrid Structures: Principles and applications.

[7] Amancio-Filho S., Beyer M., Santos J. (2009). Method for Connecting a Metallic Bolt to a Plastic Piece. U.S. Patent.

[8] Amancio-Filho S.T., dos Santos J.F. (2011). Henry Granjon Prize Competition 2009 Winner Category A: “Joining and Fabrication Technology” Friction Riveting: Development and Analysis of a New Joining Technique for Polymer-Metal Multi-Material Structures. Weld. World.

[9] Amancio-Filho S.T., Dos Santos J.F. FricRiveting: A new technique for joining thermoplastics to lightweight alloys. Proceedings of Annual Technical Conference of the Society of Plastic Engineers—ANTEC.

[10] Rodrigues C.F., Blaga L.A., dos Santos J.F., Canto L.B., Hage E., Amancio-Filho S.T. (2014). FricRiveting of aluminum 2024-T351 and polycarbonate: Temperature evolution, microstructure and mechanical performance. J. Mater. Process. Technol..

[11] Blaga L., Bancilă R., dos Santos J.F., Amancio-Filho S.T. (2013). Friction Riveting of glass–fibre-reinforced polyetherimide composite and titanium grade 2 hybrid joints. Mater. Des..

[12] Borges M.F. (2013). Desenvolvimento de nova geometria de rebite para uso em estruturas híbridas compósito-metal obtidas através do processo de rebitagem por fricção. Ph.D. Thesis.

[13] Wirth J.G., Kirshenbaum G.S. (1986). Discovery and Development of Polyetherimides. High Performance Polymers: Their Origin and Development.

[14] Johnson R.O., Burlhis H.S. (2007). Polyetherimide: A new high-performance thermoplastic resin. J. Polym. Sci. Polym. Symp..

[15] Fukuhara M. (2003). Temperature dependency of elastic moduli and internal dilational and shear frictions of polyetherimide. J. Appl. Polym. Sci..

[16] Thomas S., Visakh P.M. (2012). Handbook of Engineering and Specialty Thermoplastics.

[17] (2011). Duratron U1000 PEI; Quadrant Plastics. https://www.quadrantplastics.com/de/produkte/technische-kunststoffe/temperatur-160-220-c/duratronr-pei/?r=1.

[18] Military Handbook—MIL-HDBK-5H: Metallic Materials and Elements for Aerospace Vehicle Structures. https://app.knovel.com/web/toc.v/cid:kpMHMILH61/viewerType:toc.

[19] (2009). Metallic Materials—Tensile Testing—Part 1: Method of Test at Room Temperature.

[20] Ma T.J., Li W., Yang S.Y. (2009). Impact toughness and fracture analysis of linear friction welded Ti–6Al–4V alloy joints. Mater. Des..

[21] Crawford R.J., Tam Y. (1981). Friction welding of plastics. J. Mater. Sci..

[22] Montgomery D.C. (2001). Design and Analysis of Experiments.

[23] Myers R.H., Montgomery D.C., Anderson-Cook C.M. (1995). Response Surface Methodology: Process and Product Optimization Using Designed Experiments, 3rd ed.

[24] Amancio-Filho S.T. (2007). Friction Riveting: Development and analysis of a new joining technique for polymer-metal multi-materials structures. Ph.D. Thesis.

[25] Altmeyer J., dos Santos J.F., Amancio-Filho S.T. (2014). Effect of the friction riveting process parameters on the joint formation and performance of Ti alloy/short-fibre reinforced polyether ether ketone joints. Mater. Des..

[26] Montgomery D.C., Runger G.C. (2003). Applied statistics and probability for engineers.

[27] Amancio-Filho S.T., Dos Santos J.F. Preliminary analytical modeling of heat input in friction riveting. Proceedings of the Annual Technical Conference—ANTEC.

